# ^68^Ga-citrate visualization study on ferroptosis through transferrin receptor 1 in periodontitis

**DOI:** 10.1186/s12903-026-09237-y

**Published:** 2026-07-13

**Authors:** Yeungyeung Liu, Yingxin Li, Bingyu Ran, Xiaoling Cao, Qijun Cai, Yong Cheng, Bin Guo, Jian Gong, Jingjie Shang, Hao Xu

**Affiliations:** 1 Department of Periodontology, Stomatological Hospital, School of Stomatology, Southern Medical University, Guangzhou, Guangdong 510280 China; 2 Department of Nuclear Medicine, The First Affiliated Hospital of Jinan University, Guangzhou, Guangdong 510630 China

**Keywords:** ^68^Ga-citrate, Periodontitis, Ferroptosis, TfR1, Molecular imaging

## Abstract

**Background:**

The loss of alveolar bone in periodontitis is linked to ferroptosis mediated by Transferrin Receptor 1 (TfR1). This study employed a multimethod approach integrating in vitro and in vivo experiments to investigate the utility of gallium-68 citrate (^68^Ga-citrate), a high-affinity iron-mimetic tracer, for the non-invasive, dynamic positron emission tomography/computed tomography (PET/CT) imaging of ferroptosis and periodontal remodeling in vivo.

**Methods:**

In vitro, MC3T3-E1 were stimulated with *Porphyromonas gingivalis* lipopolysaccharide (LPS) to model the inflammatory microenvironment. The correlation between TfR1 expression and ^68^Ga-citrate uptake was validated via cellular uptake assays and plasmid-mediated TfR1 knockdown. Ferroptosis markers - TfR1, glutathione peroxidase 4 (GPX4), interleukin-1β (IL-1β), and osteocalcin (OCN), were analyzed through Western blotting (WB), quantitative PCR (qPCR) and immunohistochemistry (IHC) to characterize the ferroptotic and osteogenic profiles within periodontal tissues. In vivo, a ligature-induced periodontitis rat model was established and treated with minocycline. Periodontal inflammation and bone metabolism were longitudinally evaluated using multi-modal PET/CT ^68^Ga-citrate, 2-deoxy-2-[^18^F]fluoro-D-glucose (^18^F-FDG), and[^18^F]sodium fluoride (^18^F-NaF), complemented by micro-computed tomography (micro-CT).

**Results:**

In vitro, LPS dose-dependently reduced MC3T3-E1 viability while upregulating TfR1 and IL1β, and downregulating GPX4 expression. ^68^Ga-citrate uptake correlated positively with TfR1 expression and was significantly inhibited by TfR1 knockdown. In vivo, ^68^Ga-citrate SUV values in periodontal lesions increased progressively from day 7 to 14, accompanied by upregulation of TfR1 and bone resorption. Conversely, minocycline treatment significantly suppressed TfR1 expression and attenuated ^68^Ga-citrate SUV compared to periodontitis groups. The presence of active inflammation and impaired calcium metabolism were confirmed by conventional tracers ^18^F-FDG and ^18^F-NaF PET/CT.

**Conclusion:**

Osteoblast exhibited increased ^68^Ga-citrate uptake under inflammatory conditions, and ^68^Ga-citrate PET/CT imaging demonstrates enhanced uptake in periodontitis. The expression of TfR1 in both osteoblast and periodontitis animal models is consistent with gallium absorption. These findings indicate that ^68^Ga-citrate PET/CT can reflect ferroptosis-associated molecular alterations by targeting TfR1.

**Supplementary Information:**

The online version contains supplementary material available at https://doi.org/10.1186/s12903-026-09237-y.

## Background

Periodontitis is a chronic infectious disease affecting the tooth-supporting tissues, ultimately leading to periodontal attachment loss and gingival recession [1–3]. The initiation and advancement of periodontitis are closely related to abnormalities in alveolar bone metabolism. Infected periodontal tissues consistently exhibit an upregulation of iron-containing compounds, including ferritin, transferrin, and heme [4–6]. Recent studies have highlighted the association between ferroptosis and periodontitis, indicating that this iron-dependent form of cell death is actively involved in disease progression [7].

Within this inflammatory microenvironment, the elevated or dysregulated expression of TfR1 serves as a critical trigger for ferroptosis under inflammatory conditions. Wang et al. reported that periodontal ligament stem cells (PDLSCs) stimulated with *Fusobacterium nucleatum* exhibited increased mRNA and protein expression of proinflammatory cytokines along with elevated TfR1 levels [8]. Qiao et al. demonstrated that human gingival fibroblasts stimulated with *Porphyromonas gingivalis* (*P. gingivalis*) lipopolysaccharide (LPS) showed upregulated proinflammatory cytokines and TfR1 expression, accompanied by reduced intracellular glutathione (GSH) [9]. Similarly, in vivo studies using periodontitis mouse models have revealed the downregulation of anti-ferroptotic markers (e.g., GPX4, Nrf2, and NADPH) alongside the upregulation of SLC7A11 and TfR1. This is accompanied by an exacerbated inflammatory profile characterized by increased IL6, IL1β, and IL8, and decreased IL10. The administration of ferroptosis inhibitors reversed these molecular alterations and significantly alleviated the inflammatory response in periodontal tissues [9, 10].

These findings indicated that the upregulation of TfR1 induced by LPS was a vital factor in initiating ferroptosis. It has long been recognized that Gallium ions compete with iron ions for binding sites on transferrin, and studies have shown that Gallium ions can induce ferroptosis [11]. Meanwhile, in isotope research, ^68^Ga-labeled peptides have been used in preclinical PET imaging studies of TfR1 in tumors [12]. ^68^Ga-citrate is a radiotracer that acts as an iron mimetic in iron metabolism. It can bind to bacterial ferritin and neutrophil lactoferrin, or be directly absorbed by siderophores with a high affinity for gallium, facilitating its distribution in the circulatory system [13]. In skeletal inflammatory diseases, ^68^Ga-citrate PET/CT has demonstrated promising clinical utility, offering high overall accuracy and reliable negative predictive value. ^68^Ga-citrate PET/CT has been shown to be effective in accurately differentiating between infectious and aseptic inflammation [14]. However, studies specifically investigating the relationship between ^68^Ga-citrate and ferroptosis itself remain scarce.

Therefore, this study utilized ^68^Ga-citrate PET/CT to non-invasively visualize the ferroptotic process in periodontitis by targeting TfR1. We hypothesized that this dynamic molecular imaging platform would provide deeper insights into the complex in vivo dynamics of periodontal inflammation and alveolar bone remodeling.

## Methods

### Cell culture and treatment

MC3T3-E1 Subclone 14 was acquired from Shanghai QuiCell Biotechnology. The cells were grown in α-minimum essential medium (α-MEM, Gibco, Thermo Fisher Scientific, Carlsbad, CA, USA) with 10%fetal bovine serum (FBS, Gibco) in a humidified environment containing 5% CO_2_ at 37 °C. In preparation for subsequent experiments, cells were plated in 6-well plates at density of 2 × 10^5^ cells per well. Cell viability was assessed using CCK-8 assay after treatment with different concentrations of LPS (1, 5, 10, 20, 40 and 80 mg/mL).

### DCFH-DA Reactive Oxygen Species (ROS) assay

MC3T3-E1 cells were plated in 24-well plates and incubated until they reached logarithmic growth phase .Intracellular ROS levels were assessed using DCFH-DA probe diluted in PBS to create a 10 mmol/L working solution .The culture medium was removed, and the cells were rinsed twice with PBS. And 1 mL of DCFH-DA working solution was added to each well, and cells were incubated in the dark at 37 °C for 20-30 min. Following incubation, the cells were washed three times with PBS to eliminate any excess extracellular probes. Fluorescence was detected under a fluorescence microscope (excitation: 488 nm; emission: 525 nm).

### TfR1 interference fragment plasmid transfection

The transcript variants and CDS region of the murine TfR1 gene were acquired from the NCBI database, an additional table file shows this in more detail [see Additional file 1]. The interference plasmid was constructed using SnapGene software. The pEGFP-C1 vector was cleaved with Bgl II and EcoR I, while the pCMV-3 × FLAG-N vector was cut using BamH I. When MC3T3-E1 cells reached 70–80% confluence, the culture medium was replaced with fresh α-MEM. For transfection in a 6-well plate, a mixture containing 125 µL of MEM, 4 µL of Lipo8000™ transfection reagent, and 2 µg of plasmid DNA was prepared per well. The prepared transfection complex was thoroughly mixed, incubated at room temperature, and then added slowly to each well. The plate was gently rotated and allowed to incubate for 48 h. The transfection efficiency was confirmed, the medium was replaced, and subsequent experiments were performed after an additional 24 h of culture.

### Cellular uptake assay

MC3T3-E1 cells were plated in 12-well culture plates at density of 4 × 10^5^ cells per well. Following a 24-hour incubation period, cells were treated with 10 µg/mL LPS for another 24 h. The viable cells in designated counting wells were quantified to establish a baseline.The culture medium was subsequently substituted with glucose-free α-MEM that included ^68^Ga-citrate at concentration of 1 µCi/mL. The cells were cultured for 30 min and 60 min, with three replicate wells per time point. The radioactive material was disposed of, and the cells were rinsed three times with cold PBS. Then 1 mL of 1 mol/L NaOH solution was added to each well to completely lyse the cells. The lysate from each well was gathered and the radioactivity was assessed using a gamma counter.

### Western blot assay

Western blotting was performed as previously described [15]. The primary antibodies used in this assay included anti-TfR1 (ab269513, Abcam, Cambridge, UK), anti-GPX4 (ab282533, Abcam), and anti-IL1β (ab315084, Abcam).

### Quantitative Polymerase Chain Reaction (qPCR)

According to earlier report [16], total RNA was extracted from tissues and cultured cells using TRIzol reagent (Invitrogen, Carlsbad, CA, USA) in accordance with the manufacturer’s guidelines. Complementary DNA (cDNA) was generated using Reverse Transcription Kit from Takara (Dalian, China). qPCR was conducted using SmartCycler^®^ II System (Cepheid Inc., Sunnyvale, CA, USA), with GAPDH used as internal reference gene. The relative quantification of mRNA transcripts was calculated using the 2^ΔΔ^Ct method.

### Ligature-induced periodontitis model in rats

Thirty specific-pathogen-free (SPF) male Sprague-Dawley rats (200–300 g, 8 weeks old) were obtained from the Guangzhou Experimental Animal Center. All animal experiments were approved by the Laboratory Animal Welfare and Ethics Committee of Jinan University (Approval No.20251209-04). All animals were housed in plastic cages under standard conditions with a standard pellet diet and tap water ad libitum under a 12-h light/dark cycle. Rats were weighed and anesthetized via continuous inhalation of 3% isoflurane using a small animal gas anesthesia system, followed by supine positioning. The periodontitis model was established based on previously described protocols [17, 18]. The rats were randomly assigned to five groups (*n* = 6 per group): a control group (Con) consisting of untreated healthy rats; an experimental periodontitis group subjected to ligature-induced periodontitis for 7 days (EP7); an experimental periodontitis group subjected to ligature-induced periodontitis for 14 days (EP14); and two minocycline therapy groups treated for 7 days (MN7) and 14 days (MN14) post-ligation. For the MN7 and MN14 groups, 0.2 mg of minocycline hydrochloride ointment (Periocline, 2%; Sunstar, Japan) was locally applied into the gingival crevices once a week. Following the experimental period, rats were humanely euthanized via an intraperitoneal injection of sodium pentobarbital (150 mg/kg) to induce deep anesthesia. Upon confirmation of complete unconsciousness and loss of reflexes, cervical dislocation was performed as a secondary method to ensure humane termination.

### Micro-Computed Tomography (Micro-CT) analysis

Following euthanasia, the maxillae of the rats were dissected and scanned using a Venus Micro-CT scanner (90 kV, 80 mA) at a scanning resolution of 12 μm. The furcation region of maxillary second molar was designated as region of interest (ROI). Three-dimensional images were reconstructed using Cruiser and Recon software, and bone volume (BV) and tissue volume (TV) were measured using Avatar software to calculate the BV/TV fraction. Additionally, the linear distance from the cementoenamel junction (CEJ) to the alveolar bone crest (ABC) was measured at both the mesial and distal aspects of the maxillary second molar, and the average value was recorded.

### PET/CT imaging

The ^68^Ga-citrate t racer was purchased by the Atomic High-Tech Co., Ltd (Guangzhou, China). ^18^F-NaF and ^18^F-FDG were provided by the Department of Nuclear Medicine, The First Affiliated Hospital of Jinan University, with radiochemical purity > 99%. The IRIS small-animal PET/CT imaging system (Inviscan SAS, Strasbourg, France) was utilized to conduct PET/CT imaging. During the scanning procedure, the rats were anesthetized with 3% isoflurane, and their body temperature was maintained at 37.5 °C using a temperature regulation unit (Minerve équipement vétérinaire, Marne, France). For tracer injection and scanning: ^68^Ga-citrate PET/CT: After intravenous injection of ^68^Ga-citrate (15–20 MBq) via the tail vein, a 10-minute static PET scan was acquired starting 45 min post-injection. ^18^F-FDG PET/CT: After intravenous injection of ^18^F-FDG (15–20 MBq) via the tail vein, a 10-minute static PET scan was acquired starting 60 min post-injection. ^18^F-NaF PET/CT: After intravenous injection of ^18^F-NaF (15–20 MBq) via the tail vein, a 10-minute static PET scan was acquired starting 60 min post-injection. The PET data were reconstructed utilizing 3D Ordered Subsets Expectation Maximization (3D-OSEM) algorithm that incorporates a Monte Carlo-based accurate detector model. Following the PET scan, a CT scan was conducted with the X-ray tube voltage set at 80 kV and the current at 0.9 mA. PET images were analyzed using PMOD 4.1 software (PMOD Technologies, Zurich, Switzerland). Bilateral regions of interest (ROIs) were manually delineated in the maxillary region, extending from the mesial aspect of the first molar to the distal aspect of the third molar. The average standardized uptake value (SUV) within the ROI was calculated.

### Immunohistochemistry

Immunohistochemical staining for TfR1 and OCN was conducted on sections of paraffin-embedded maxilla. Sections were deparaffinized, rehydrated, and treated with 3% hydrogen peroxide for 25 min at room temperature. Following blocking with bovine serum albumin (BSA) for 30 minutesat room temperature, sections were incubated with primary antibodies against TfR1 (ab269531, 1:200; Abcam, Cambridge, UK) and OCN (GB115684- 100, 1:500; Servicebio, Wuhan, China) overnight at 4 °C. Subsequently, the sections were treated with biotinylated secondary antibody (HRP-labeled goat anti-rabbit IgG, 1:200; Servicebio) for 50 min at room temperature. The slides were washed three times in PBS (pH 7.4 ) for 5 min each. A freshly prepared DAB chromogenic solution was applied for signal detection, followed by counterstaining with hematoxylin for 3 min. The sections were dehydrated in a sequential manner using a graded series of alcohol 75% for 5 min, 85% for 5 min, absolute ethanol twice for 5 min each, and n-butyl alcohol for 5 min, followed by a clearing step in xylene for 5 min. Ultimately, the sections were briefly air-dried and placed in a mounting medium. Images were taken with a light microscope (Leica DM6b, Nussloch, Germany) and analyzed through ImageJ software (NIH, Bethesda, MD, USA). The same cohort of animals underwent longitudinal multimodal PET/CT imaging (in vivo) and, after sacrifice, the maxillae were harvested for micro-CT and histological analyses.

### Statistical analysis

All statistical analyses were performed using GraphPad Prism 8 software (GraphPad Software, San Diego, CA, USA). Data are presented as the mean ± standard deviation (SD) derived from at least three independent experiments. Group comparisons were assessed using Student’s t-test (for two groups) or one-way analysis of variance (ANOVA) followed by appropriate post hoc tests (for multiple groups). A P-value of < 0.05 was considered statistically significant.

## Results

### Osteoblast ferroptosis occurs under inflammatory conditions

**Fig. 1:**
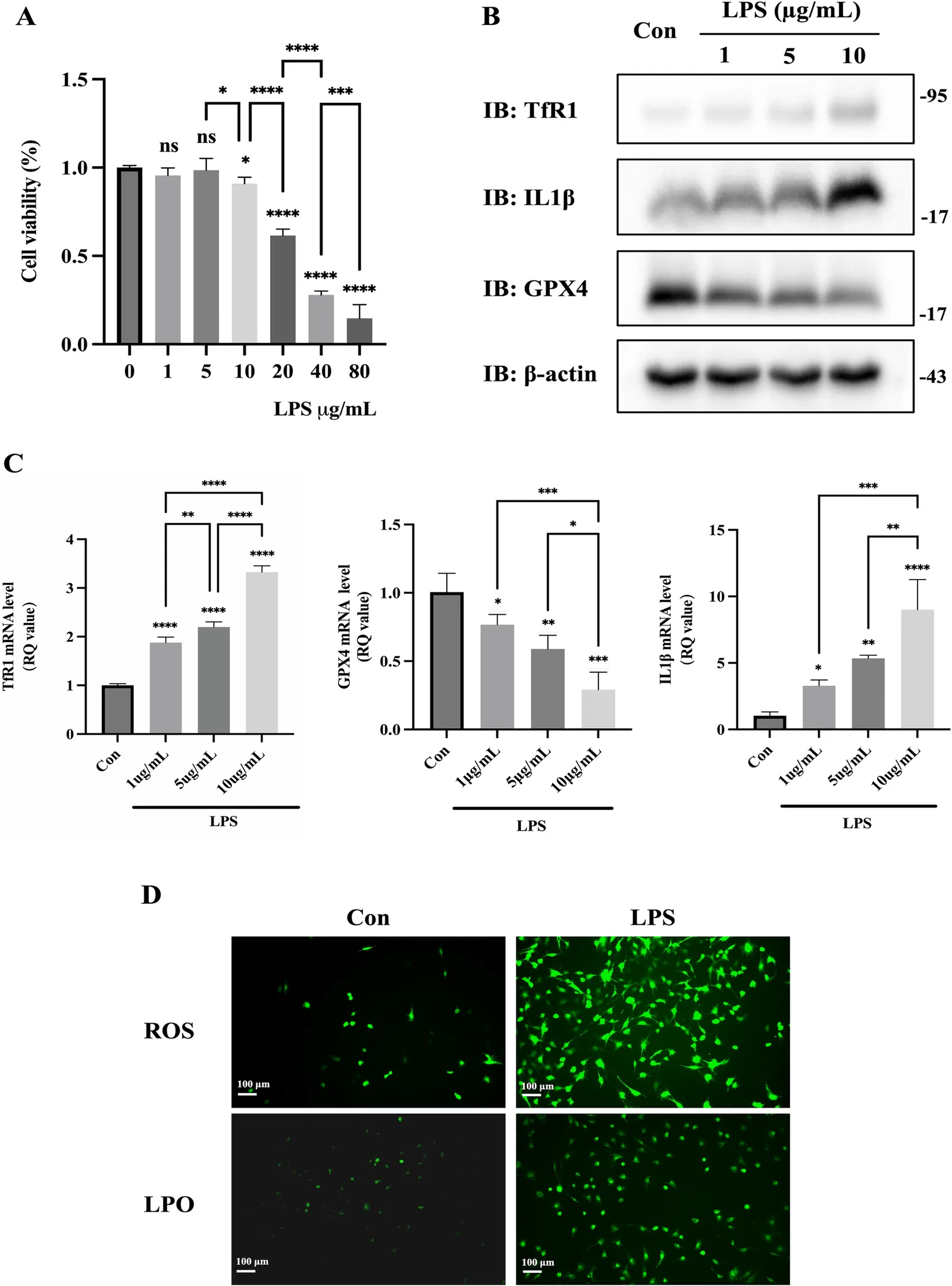
Osteoblast ferroptosis occurred at inflammatory status. **A** The cck8 assay was performed after MC3T3 cells treated with LPS (1 µg/mL, 5 µg/mL, and 10 µg/mL). **B** Western blot analysis was performed to detect the expression of TfR1, IL1β, and GPX4 proteins after intervention with LPS in MC3T3 cells. **C** The qPCR was performed to detect the RNA transcription of TfR1, IL1β, and GPX4 after MC3T3 cells were treated with LPS. **D** DCFH-DA reactive oxygen species (ROS) was performed after MC3T3 cells were treated with 10 µg/mL LPS. *: *P* < 0.05, **: *P* < 0.01, ***: *P* < 0.001, ****: *P* < 0.0001, ns: not significant

To evaluate the impact of the inflammatory microenvironment on ferroptosis, MC3T3-E1 cells were incubated with LPS for 24 h, followed by the assessment of ferroptosis biomarkers. As demonstrated in Fig. 1A, LPS dramatically reduced the viability of MC3T3-E1 cells in a dose-dependent manner, with a notable decrease seen at 10 µg/mL. Furthermore, the expression of TfR1 and IL1β gradually increased, whereas GPX4 expression decreased, in response to escalating LPS concentrations (0, 1, 5, and 10 µg/mL) (Fig. 1B). A consistent trend was observed in the mRNA transcription levels of TfR1, IL1β, and GPX4 across the corresponding LPS concentrations (Fig. 1C). At the level of 10 µg/mL LPS, cell ROS and LPS accumulation was increased (Fig. 1D).

### ^68^Ga-citrate reflects osteoblast ferroptosis via TfR1

**Fig. 2:**
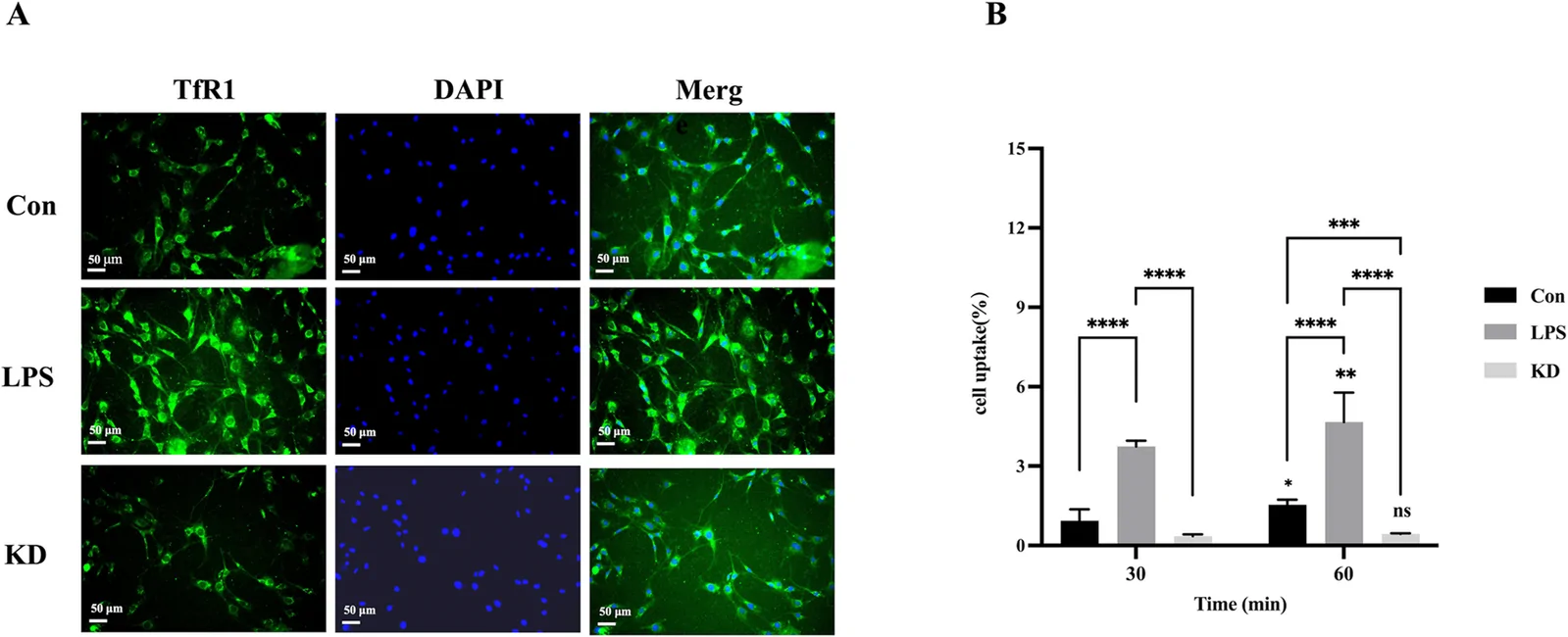
^68^Ga-citrate reflexed osteoblast ferroptosis through TfR1. **A** MC3T3 cells were treated with 10 µg/ mL LPS in LPS group. TfR1 immunofluorescence staining was observed in Control group (Con), Knocked Down group (KD) and LPS group (LPS). **B** Perform ^68^Ga-citrate uptake experiments on MC3T3 cells in each group and record the cell uptake ratio. *: *P* < 0.05, **: *P* < 0.01, ***: *P* < 0.001, ****: *P* < 0.0001, ns: not significant

Because ^68^Ga-citrate serves as an imaging surrogate for iron metabolism, and TfR1 is a pivotal protein in this pathway, we evaluated ^68^Ga-citrate cellular uptake following plasmid-mediated TfR1 knockdown (KD) in MC3T3-E1 cells, an additional figure file shows this in more detail [see Additional file 2]. Compared to cells treated with 10 µg/mL LPS alone, cells transfected with the TfR1-KD plasmid exhibited a significant reduction in TfR1 protein expression. Subsequent cellular uptake assays were performed at 30 and 60 min post-^68^Ga-citrate administration. The tracer uptake rate was considerably higher in the LPS-treated group compared to the Con group at both time points. Conversely, the KD group demonstrated the lowest ^68^Ga-citrate uptake rate, confirming that the tracer’s accumulation is highly dependent on TfR1 expression (Fig. 2).

### Alveolar bone defects and inflammatory changes in the periodontitis animal model

**Fig. 3:**
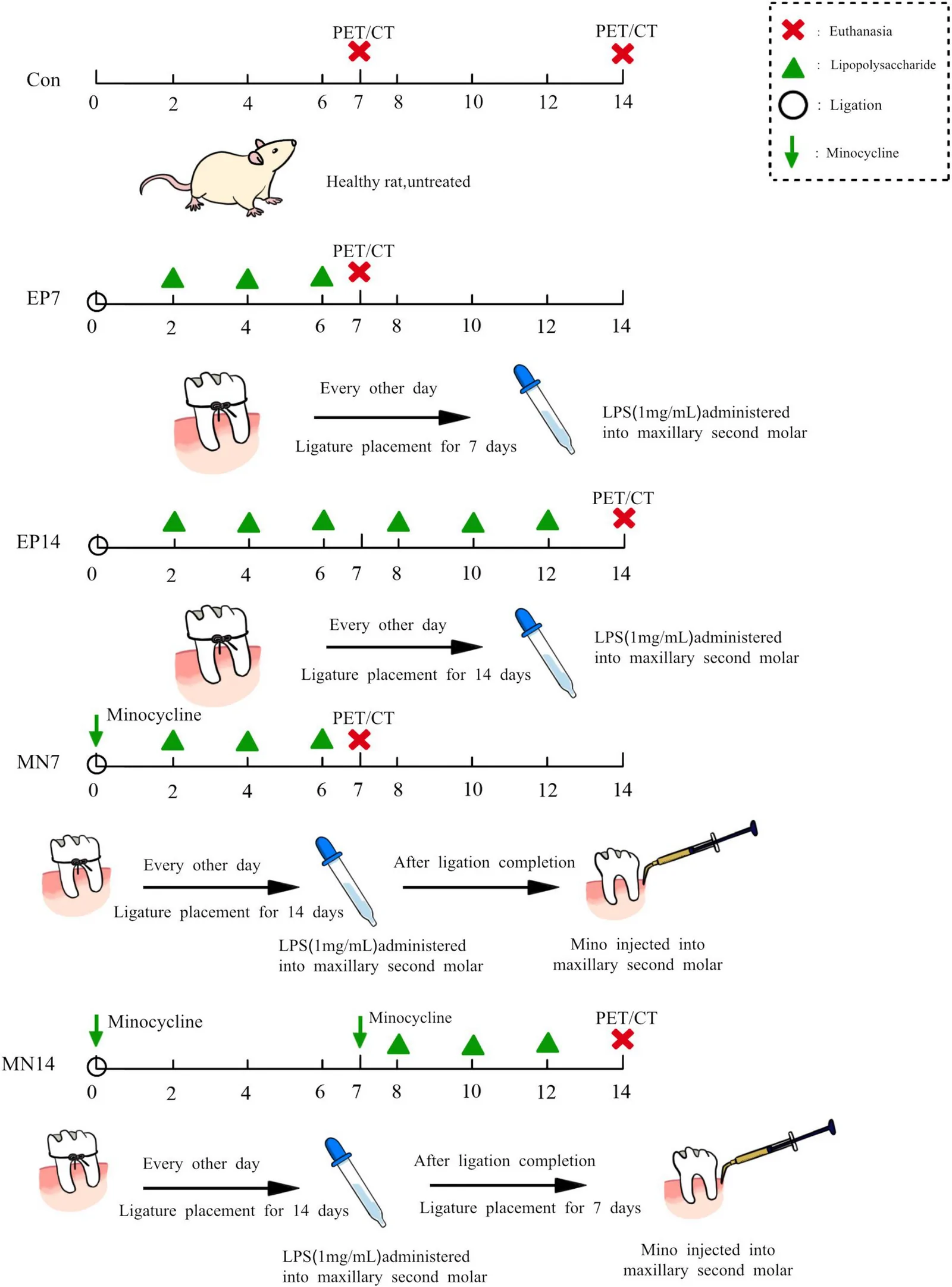
Ligature-induced periodontitis model in rats. A Control group (Con) consisting of untreated healthy rats; an experimental periodontitis group subjected to ligature-induced periodontitis for 7 days (EP7); an experimental periodontitis group subjected to ligature-induced periodontitis for 14 days (EP14); and two minocycline therapy groups treated for 7 days (MN7) and 14 days (MN14) post-ligation. For the MN7 and MN14 groups, 0.2 mg of minocycline hydrochloride ointment (Periocline, 2%; Sunstar, Japan) was locally applied into the gingival crevices once a week

**Fig. 4:**
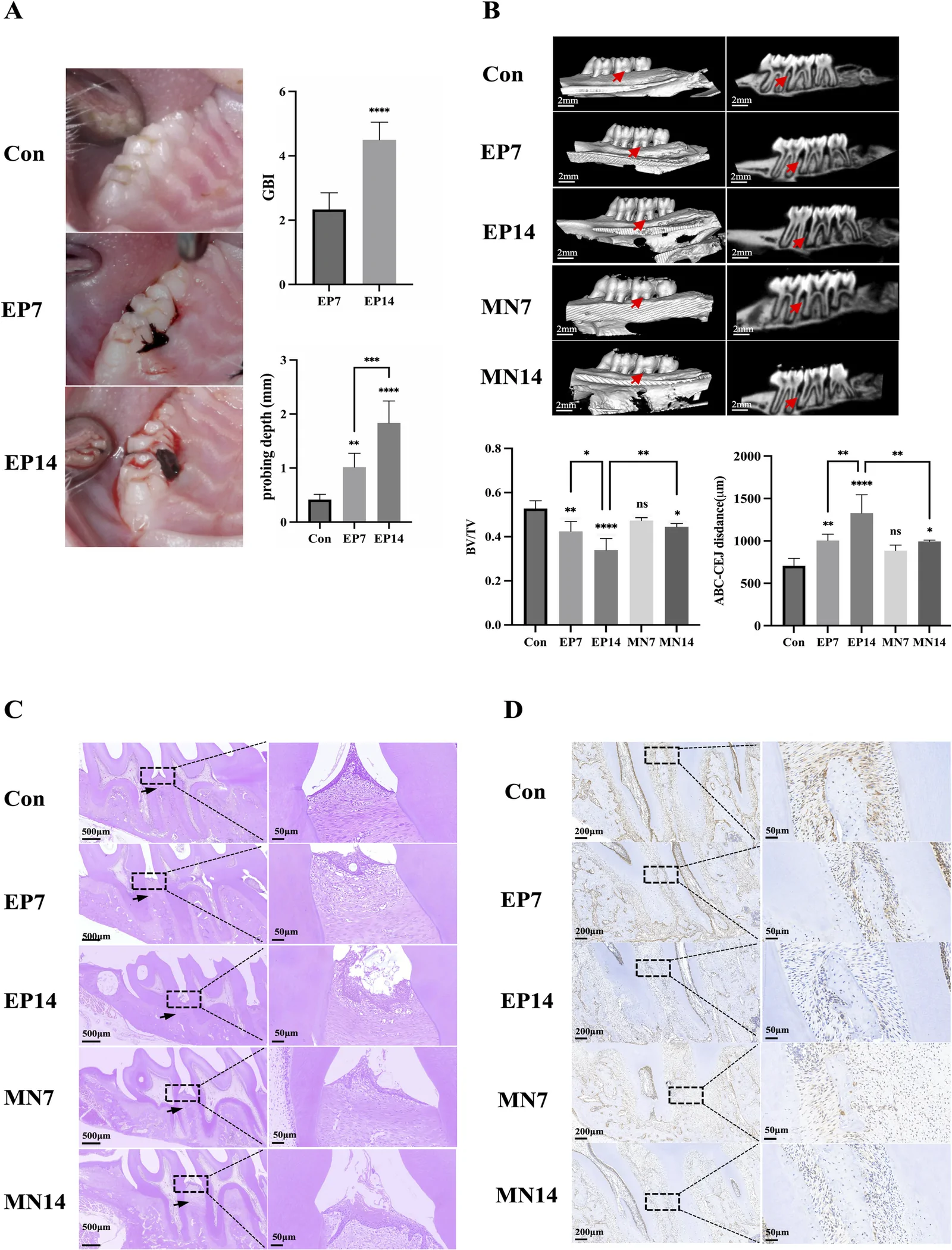
Bone defect occured in periodontitis animal model. A periodontitis rat model was established. Ligature at 7 days post-modeling was designated as EP7 group (EP7), and Ligature at 14 days post-modeling was designated as EP14 group (EP14). The minocycline group (Mino group) received intragingival injection of minocycline hydrochloride ointment into the gingiva of the second molar of the maxilla. The group receiving minocycline at 7 days post-modeling was designated as Mino7 group (MN7), and the group receiving minocycline at 14 days post-modeling was designated as Mino14 group (MN14). **A** GI index was evaluated. **B **Three-dimensional Micro-CT images and sagittal tomographic images of the maxillary bones in each group with CEJ-ABC distances, red arrows indicate the alveolar bone height. **C** HE staining of alveolar tissue in the ligated model of periodontitis rats. **D** OCN staining of alveolar tissue in the ligated model of periodontitis rats. **: *P* < 0.01, ***: *P* < 0.001, and ****: *P* < 0.0001, ns: not significant

**Fig. 5:**
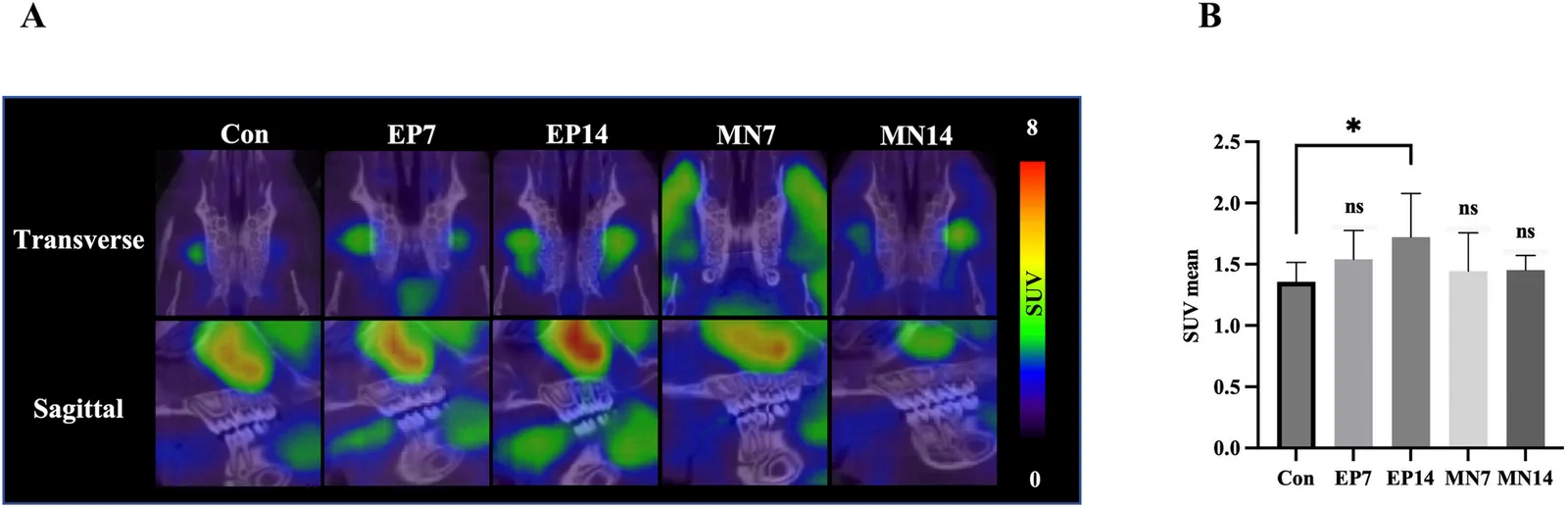
Changes of ^18^F-FDG PET/CT in periodontitis animal model. ^18^F-FDG PET/CT: After intravenous injection of ^18^F-FDG (15–20 MBq) via the tail vein, a 10-minute static PET scan was acquired starting 60 min post-injection. **A **^18^F-FDG PET/CT scans were performed at corresponding time points to measure the mean SUV values of ^18^F-FDG PET/CT in rats across all groups. **B** Statistical analysis was performed on the mean ^18^F-FDG PET/CT SUV values of all rat groups. *: *P* < 0.05, ns: not significant

**Fig. 6:**
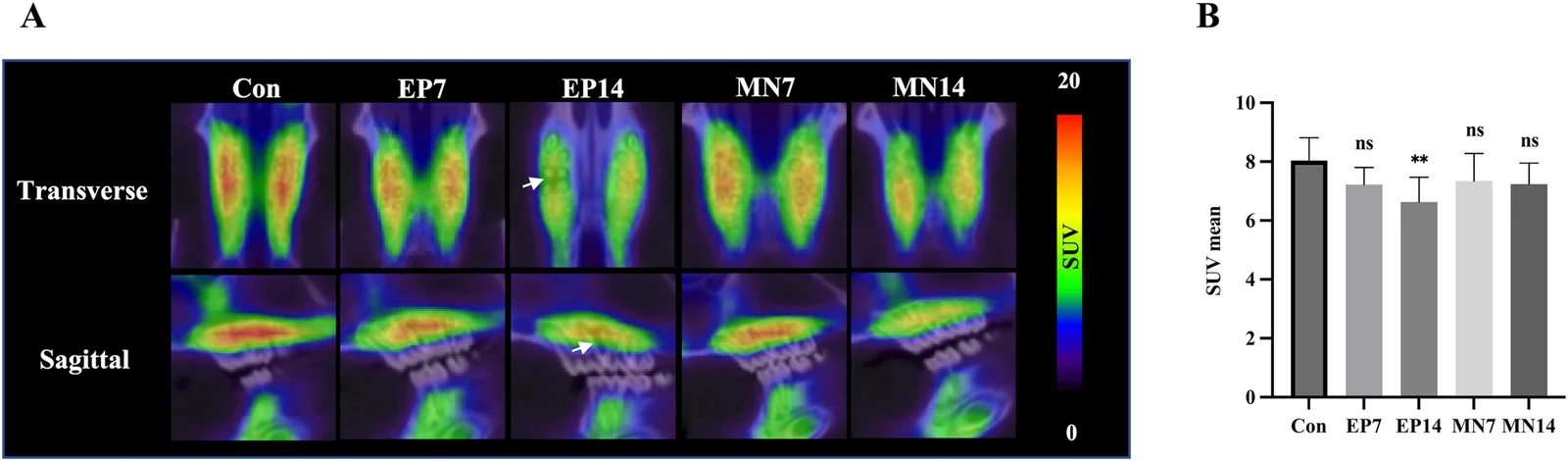
Changes of ^18^F-Na PET/CT in periodontitis animal model. ^18^F-NaF PET/CT: After intravenous injection of ^18^F-NaF (15–20 MBq) via the tail vein, a 10-minute static PET scan was acquired starting 60 min post-injection. **A** ^18^F-Na PET/CT scans were performed at corresponding time points to measure the mean SUV values of ^18^F-Na PET/CT in rats across all groups.The white arrows point to the site of alveolar bone resorption at the maxillary second molar, characterized by decreased ^18^F-NaF accumulation reflecting impaired calcium metabolism. **B** Statistical analysis was performed on the mean ^18^F-Na PET/CT SUV values of all rat groups. **: *P* < 0.01, ns: not significant

A ligature-induced periodontitis rat model was developed to evaluate ferroptosis during inflammatory bone loss (Fig. 3). Clinical manifestations of periodontitis were evident by day 7 post-ligation (EP7), characterized by moderate inflammation of the gingival margin and interdental papillae, presenting with bright red coloration, mild edema, and bleeding upon probing confined to the gingival sulcus. By day 14 (EP14), the inflammation progressed to a severe state, exhibiting a brittle gingival texture, severe edema, and spontaneous bleeding spilling beyond the gingival sulcus (Fig. 4A). Micro-CT analysis revealed a progressive trend of alveolar bone defects from EP7 to EP14, which was effectively attenuated by minocycline administration (Fig. 4B). Histological evaluations confirmed the presence of inflammatory infiltration and alveolar bone loss, accompanied by suppressed osteogenesis, as indicated by low OCN expression (Fig. 4C and D). Consistent with these findings, ^18^F-FDG PET/CT demonstrated intense tracer accumulation in the alveolar region under periodontitis conditions, which was subsequently reduced following antibiotic intervention (Fig. 5). Furthermore, calcium metabolism was evaluated using ^18^F-NaF PET/CT, a bone-seeking agent that binds to hydroxyapatite crystals [19]. The inflammatory environment induced a marked reduction in local calcium metabolism within the alveolar area, an effect that was partially reversed by minocycline treatment (Fig. 6).

### ^68^Ga-citrate PET/CT correlating with TfR1 upregulation and ferroptosis-associated changes in vivo

**Fig. 7:**
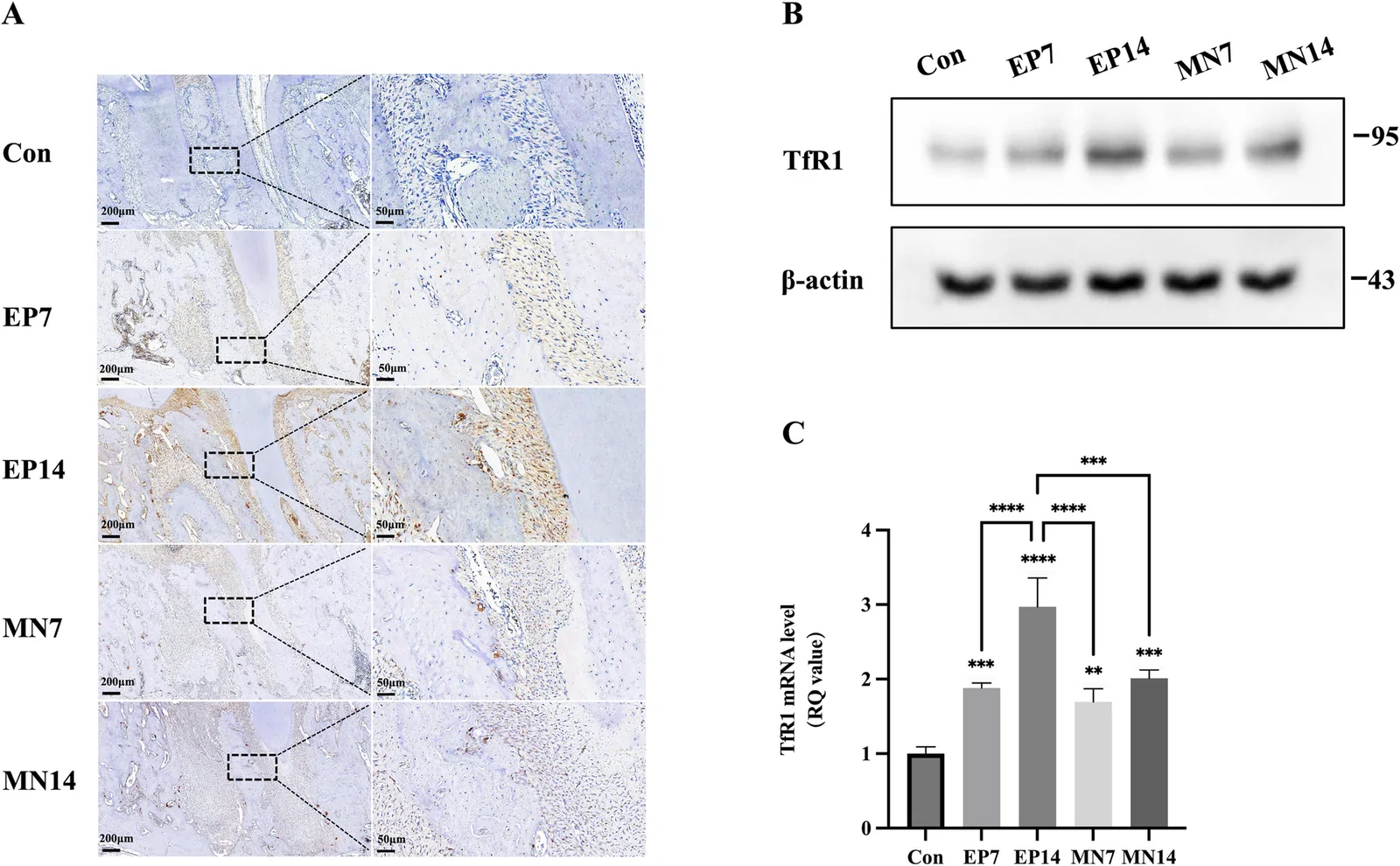
Changes in periodontitis animal models. A periodontitis rat model was established. Ligature at 7 days post-modeling was designated as EP7 group (EP7), and Ligature at 14 days post-modeling was designated as EP14 group (EP14). The Minocycline group (Mino group) received intragingival injection of minocycline hydrochloride ointment into the gingiva of the second molar of the maxilla. The group receiving minocycline at 7 days post-modeling was designated as MN7 group (MN7), and the group receiving minocycline at 14 days post-modeling was designated as MN14 group (MN14). **A** Immunohistochemical staining of TfR1 in maxillary bone tissues of rats with periodontitis. **B** Samples were collected at the corresponding time points for Western blot analysis. **C **Samples were collected at corresponding time points for qPCR analysis. **: *P* < 0.01, *** : *P* < 0.001, ****: *P* < 0.0001, ns: not significant

**Fig. 8:**
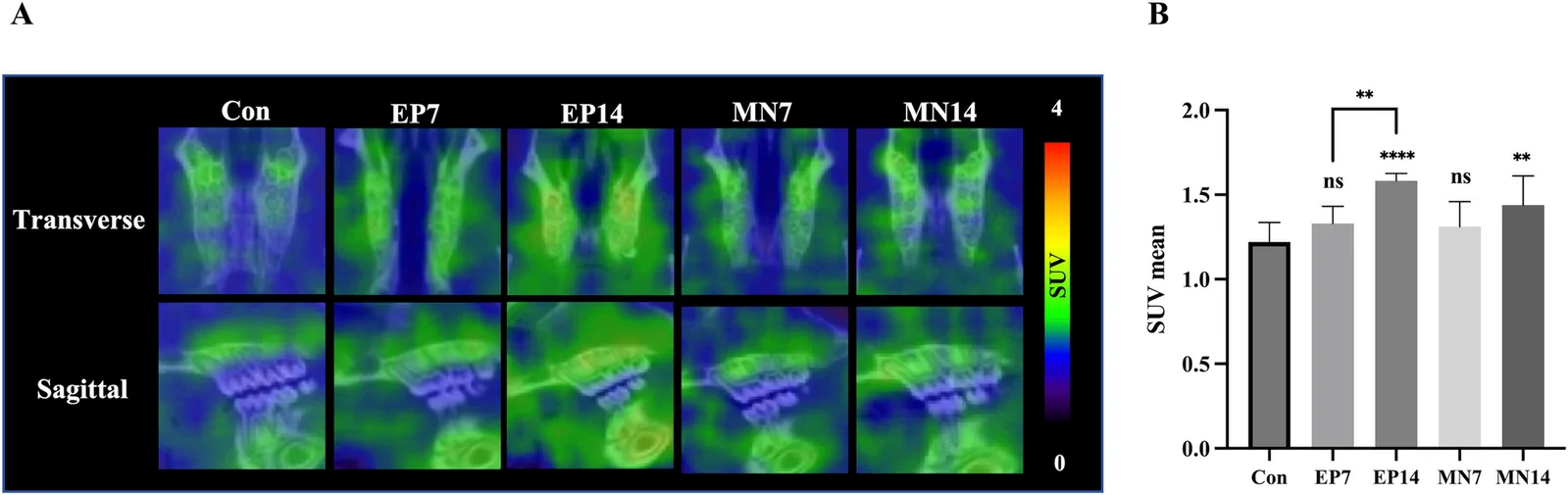
Changes of ^68^Ga-citrate PET/CT in periodontitis animal model. **A** ^68^Ga-citrate PET/CT scans were performed at corresponding time points to measure the mean SUV values of ^68^Ga-citrate PET/CT in rats across all groups. **B** Statistical analysis was performed on the mean ^68^Ga-citrate PET/CT SUV values of all rat groups. **: *P* < 0.01, ****: *P* < 0.0001, ns: not significant

In the rat periodontitis model, both the transcription and protein expression of TfR1 increased progressively alongside disease exacerbation. Notably, this inflammation-induced TfR1 upregulation was significantly inhibited by minocycline administration at matched time points (Fig. 7). In vivo PET/CT imaging acquired 45 min post-injection of ^68^Ga-citrate provided clear visualization of the maxillary bone, with minimal background uptake in the adjacent maxillary sinus and brain tissue. The uptake of ^68^Ga-citrate in the maxillae of ligature-induced periodontitis rats was markedly higher than that in the healthy control (Con) group. Quantitative analysis revealed that the mean standardized uptake values (SUV) in both the EP7 and EP14 groups were elevated compared to the Con group. Notably, a statistically significant increase in ^68^Ga-citrate SUV was observed only in the EP14 group compared to the control, whereas the EP7 group showed no significant alteration. Following minocycline intervention, the MN7 group exhibited a lower mean SUV than the EP7 group, although this trend did not reach statistical significance. In contrast, the MN14 group demonstrated a significant reduction in tracer uptake compared to the EP14 group (Fig. 8).

## Discussion

In the inflammatory microenvironment of periodontitis, ferroptosis is driven by multiple pathways that elevate intracellular ROS levels [4, 20]. The dysregulated overexpression of TfR1 serves as a critical catalyst for cellular ferroptosis during periodontal inflammation. Using an LPS-induced osteoblast inflammation model, we demonstrated that LPS intervention dose-dependently increased ROS accumulation in MC3T3-E1 cells. Concurrently, the protein expression and mRNA transcription of TfR1 and IL1β were upregulated, whereas the anti-ferroptotic enzyme GPX4 was progressively downregulated. These findings align with previous literature [21], further corroborating TfR1 as a pivotal mediator in the ferroptotic cascade. Previous studies have evidenced that ^68^Ga-citrate, a radiotracer mimicking iron metabolism, is internalized by cells via the transferrin/TfR1 complex [22]. This mechanism provides a theoretical basis for utilizing ^68^Ga-citrate to probe TfR1 expression associated with ferroptosis. In skeletal imaging, ^68^Ga-citrate has demonstrated superior specificity compared to conventional ^18^F-FDG, effectively minimizing false-negative results [23] and accurately distinguishing physiological bone healing from true osteomyelitis [24, 25]. Furthermore, it exhibits broad potential in diagnosing infectious or inflammatory diseases across various organ systems [26]. In this study, we confirmed the TfR1 dependency of ^68^Ga-citrate uptake through in vitro experimentation. Following plasmid-mediated TfR1 knockdown in MC3T3-E1 cells, the cellular uptake of ^68^Ga-citrate significantly decreased concurrently with TfR1 depletion, supporting TfR1-dependent uptake rather than establishing absolute specificity for ferroptosis.

A key pathological characteristic of periodontitis is the gradual loss of alveolar bone [27, 28]. As periodontal inflammation exacerbates, the homeostasis of alveolar bone remodeling is severely disrupted. This process is driven by multiple mechanisms: immune cell activation and release of inflammatory mediators hyperactivate osteoclasts while suppressing osteoblast function [29]; the RANKL/RANK signaling axis further amplifies the osteoclastogenesis [30]; and inflammatory stimuli trigger the secretion of matrix metalloproteinases, which degrade extracellular matrix components and impair osteoblast attachment [31, 32]. To replicate these clinical manifestations, a rat model of periodontitis was created using a ligature-induced method. Typical clinical symptoms, including gingival marginal inflammation, bright-red coloration, localized edema, and bleeding upon probing, were observed by day 7 post-ligation [33], and these symptoms significantly worsened by day 14. Micro-CT analysis revealed that the alveolar bone defects at day 14 (EP14) were significantly more severe compared to the control and day 7 (EP7) groups, as indicated by significant bone volume loss and a decrease in alveolar crest height. These in vivo phenotypes are highly consistent with the osteoblast impairment induced by LPS in vitro. Conversely, minocycline has proven efficacy in suppressing *P.gingivalis*-induced inflammatory responses clinically and reducing bone abnormalities in animal models [34, 35]. Histological analysis in our study demonstrated that as periodontitis progressed, inflammatory cell infiltration increased, while osteogenic activity (indicated by OCN deposition) markedly decreased. Following minocycline administration, both the inflammatory infiltration and the loss of calcium metabolism were significantly alleviated.

In the context of translational molecular imaging, clinical practice traditionally utilizes technetium-99m methylene diphosphonate (^99m^Tc-methylene) for labeling autologous leukocytes, alongside ^18^F-FDG, ^18^F-NaF, ^67^Ga, and ^68^Ga to visualize inflammatory diseases. Other novel targeted tracers, including 2-deoxy-2-[^18^F]fluoro-D-sorbitol(^18^F-FDS), [^18^F]fluoropropyl-aminomethylene diphosphonate (^18^F-FPTMP), and [^11^C]para-aminobenzoic acid (^11^C-PABA) also hold promise for characterizing specific microbial and inflammatory pathways [36–40]. Regarding periodontitis, while ^18^F-FDG PET/CT can monitor therapeutic responses, its lack of target specificity limits its clinical expectations [41]. Meanwhile, ^18^F-FDG PET/CT effectively tracks hydroxyapatite metabolism, indirectly reflecting alterations in periodontal bone mass. Our dual-tracer imaging using ^18^F-FDG and ^18^F-NaF successfully corroborated these findings, visualizing both the heightened inflammatory metabolic burst and the impaired bone mineral density.

However, it is important to recognize that ^68^Ga-citrate can accumulate in inflammatory and infectious lesions through mechanisms independent of TfR1. ^68^Ga-citrate binds to transferrin and lactoferrin, which are abundant in inflamed periodontal tissues, and the complex can extravasate due to increased vascular permeability, a hallmark of periodontitis. Additionally, activated neutrophils and macrophages express lactoferrin receptors that mediate cellular uptake, and certain periodontal bacteria produce siderophores with high affinity for gallium [13]. Consequently, a portion of the PET signal observed in the periodontium likely reflects these nonspecific inflammatory and infectious components, rather than solely TfR1-mediated ferroptotic activity. This raises the critical question of whether the elevated ^68^Ga-citrate uptake primarily represents general inflammatory activity instead of ferroptosis-specific biological processes. Several aspects of our data argue against the signal being entirely attributable to nonspecific inflammation. First, in vitro TfR1 knockdown significantly reduced ^68^Ga-citrate uptake in LPS-stimulated osteoblasts, indicating a substantial TfR1-dependent component. Second, the in vivo lesional uptake correlated temporally with TfR1 upregulation and bone resorption, and was suppressed by minocycline, which also downregulated TfR1. Third, our multimodal imaging strategy employed ^18^F-FDG as an internal reference for general inflammatory activity; while ^18^F-FDG highlighted the full extent of periodontal inflammation, the ^68^Ga-citrate signal exhibited a pattern that correlated more closely with ferroptosis markers than with inflammatory infiltrates alone. Nevertheless, we acknowledge that the current study cannot fully disentangle the relative contributions of TfR1-mediated ferroptosis and nonspecific inflammation to the ^68^Ga-citrate PET signal. Therefore, the imaging readout should be interpreted as a composite marker associated with ferroptosis-related molecular changes in the inflamed periodontium, rather than a definitive and specific indicator of ferroptosis per se. Future studies incorporating specific ferroptosis inhibitors or blocking agents will be necessary to isolate the TfR1-dependent signal component and validate the specificity.

As per our earlier discussion, immunohistochemical analysis confirmed that TfR1 expression in vivo escalated parallel to the severity of local inflammation and declined upon minocycline intervention. The in vivo imaging revealed a marked increase in ^68^Ga-citrate uptake within the maxillae of periodontitis rats. Interestingly, during the mild inflammatory stage, tracer uptake did not show statistically significant alterations compared to controls; marked tracer accumulation became evident only when the disease progressed to the severe stage. Following minocycline intervention, ^68^Ga-citrate PET/CT imaging captured a corresponding reduction in signal intensity, although the therapy could not completely normalize the elevated TfR1 expression to baseline levels. Collectively, these findings underscore TfR1 as a key molecular link between ferroptosis and ^68^Ga-citrate accumulation. By targeting TfR1, ^68^Ga-citrate PET/CT can reflect ferroptosis-associated molecular alterations in periodontitis, providing a novel and non-invasive functional imaging strategy for evaluating osteoblast ferroptotic changes. However, the observed PET signal likely comprises both TfR1-mediated and nonspecific inflammatory components; thus, its specificity for ferroptosis requires further validation and should not be considered definitive at this stage.

Our study has several limitations. The evidence for ferroptosis relies on surrogate markers (TfR1, GPX4, lipid peroxidation) and lacks functional validation with specific inhibitors or iron chelators, thus a definitive causal link to the ^68^Ga-citrate signal remains obscure. The tracer’s specificity for ferroptosis is also not fully established, as uptake may partially reflect nonspecific inflammation. In vitro experiments used only MC3T3-E1 pre-osteoblast line, which cannot represent the full cellular diversity of the periodontium. Moreover, the ligature-induced rat model mimics acute bone resorption but not the chronic, multifactorial pathogenesis of human periodontitis. From a translational perspective, ^68^Ga-citrate PET/CT is constrained by cost, radiation, and limited availability, precluding routine periodontal diagnosis; its potential value lies in providing functional molecular information for specific research scenarios, such as detecting early disease activity or monitoring anti-ferroptosis therapy. As a proof-of-concept study, however, clinical feasibility and cost-effectiveness require further validation before any translational application can be envisioned.

## Conclusions

In conclusion, osteoblast exhibited increased ^68^Ga-citrate uptake under inflammatory conditions, and ^68^Ga-citrate PET/CT imaging demonstrates enhanced uptake in periodontitis. The expression of TfR1 in both osteoblast and periodontitis animal models is consistent with gallium absorption. Therefore, this study suggested that ^68^Ga-citrate PET/CT can reflect ferroptosis-associated molecular alterations by targeting TfR1.

## Supplementary Information

Supplementary Material 1.

Supplementary Material 2.

Supplementary Material 3.

## Data Availability

No datasets were generated or analysed during the current study.

## Abbreviations

TfR1: Transferrin Receptor 1
Gallium-68 citrate: ^68^Ga-citrate
PET/CT: Positron emission tomography/computed tomography
GPX4: Glutathione Peroxidase 4
IL1β: Interleukin 1β
OCN: Osteocalcin
LPS: Lipopolysaccharide
ABC: Alveolar Bone Crest
BV: Bone Volume
CEJ: Cement-Enamel Junction
CT: Computed Tomography
FDG: Fludeoxyglucose
GBI: Gingival Bleeding Index
HE: Hematoxylin and eosin
LPO: Lipid peroxide
P.g: Porphyromonas gingivalis
ROI: Region Of Interest
ROS: Reactive Oxygen Species
SPECT: Single Photon Emission Computed Tomography
SUV: Standardized Uptake Value
TF: Transferrin
‌TV: Tissue Volume
KD: Knockdown
