# Supplementary material for: 68Ga-citrate visualization study on ferroptosis through transferrin receptor 1 in periodontitis
Source: BMC Oral Health. 2026 Jul 13;26:1972. doi: 10.1186/s12903-026-09237-y (PMC13656373; doi:10.1186/s12903-026-09237-y)
Supplement: Supplementary file 1 [file 12903_2026_9237_MOESM1_ESM.pdf]

Additional file 1 The transcript variants and CDS region of the TfR1 gene

Gene	Sense（5' - 3’）	Antisense（5' - 3’）
mTfr1 - 307	CCGACAAUAACAUGAAGCCUATT	UAGCCUUCAUGUUAUUGUCGGTT
mTfr1 - 1365	CCAGACCGUUAUGUUGUAGUATT	UACUACAACAUAACGGUCUGGTT
mTfr1 - 1985	CGUUGAAUUGAACCUGGACUATT	UAGUCCAGCUUCAAUUCAACGTT
