# Supplementary material for: 68Ga-citrate visualization study on ferroptosis through transferrin receptor 1 in periodontitis
Source: BMC Oral Health. 2026 Jul 13;26:1972. doi: 10.1186/s12903-026-09237-y (PMC13656373; doi:10.1186/s12903-026-09237-y)

Figure 1

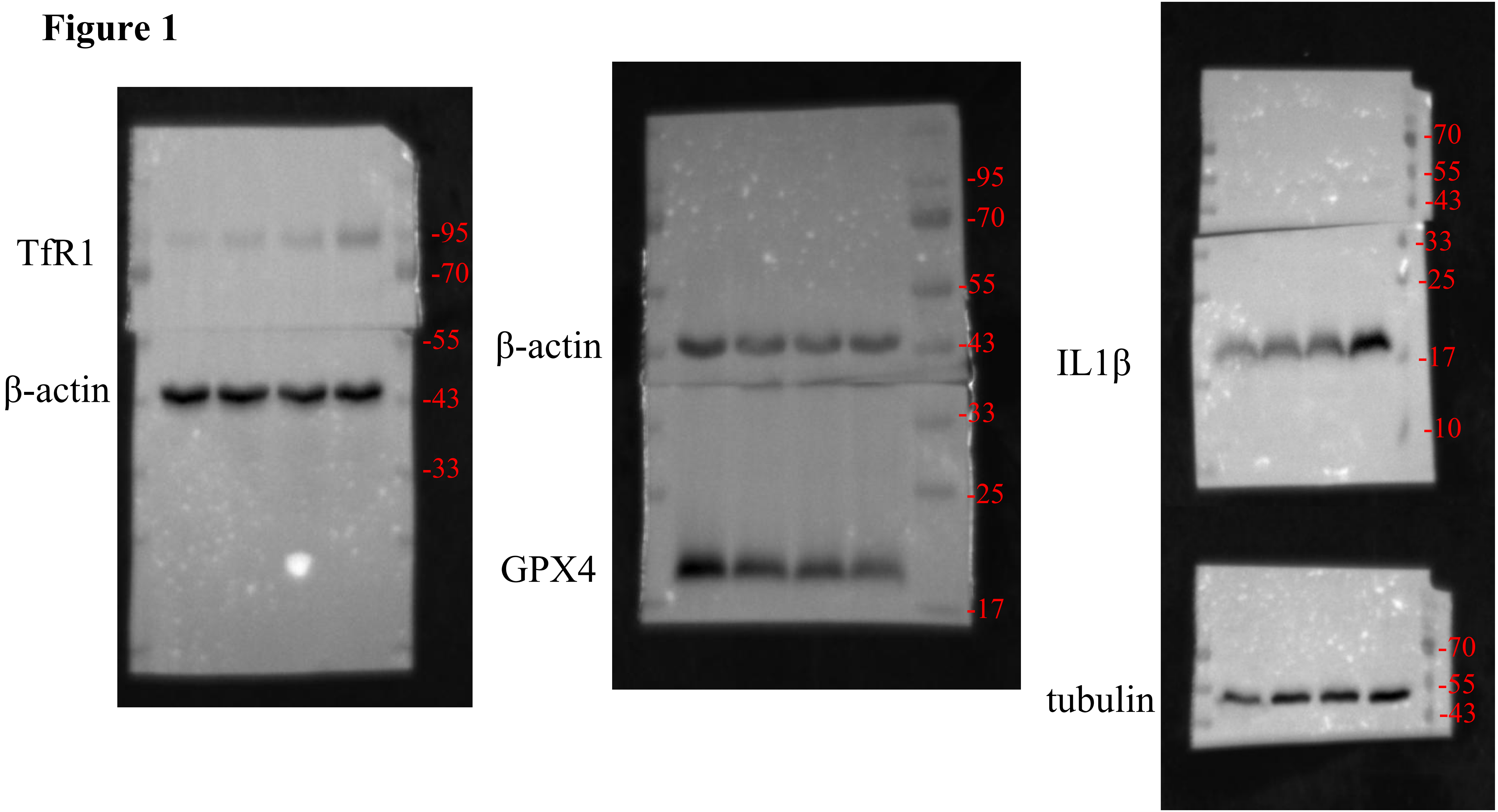

TfR1
β-actin
-95
-70
-55
-43
-33
β-actin
GPX4
-95
-70
-55
-43
-33
-25
-17
IL1β
-70
-55
-43
-33
-25
-17
-10
tubulin
-70
-55
-43

Figure 7

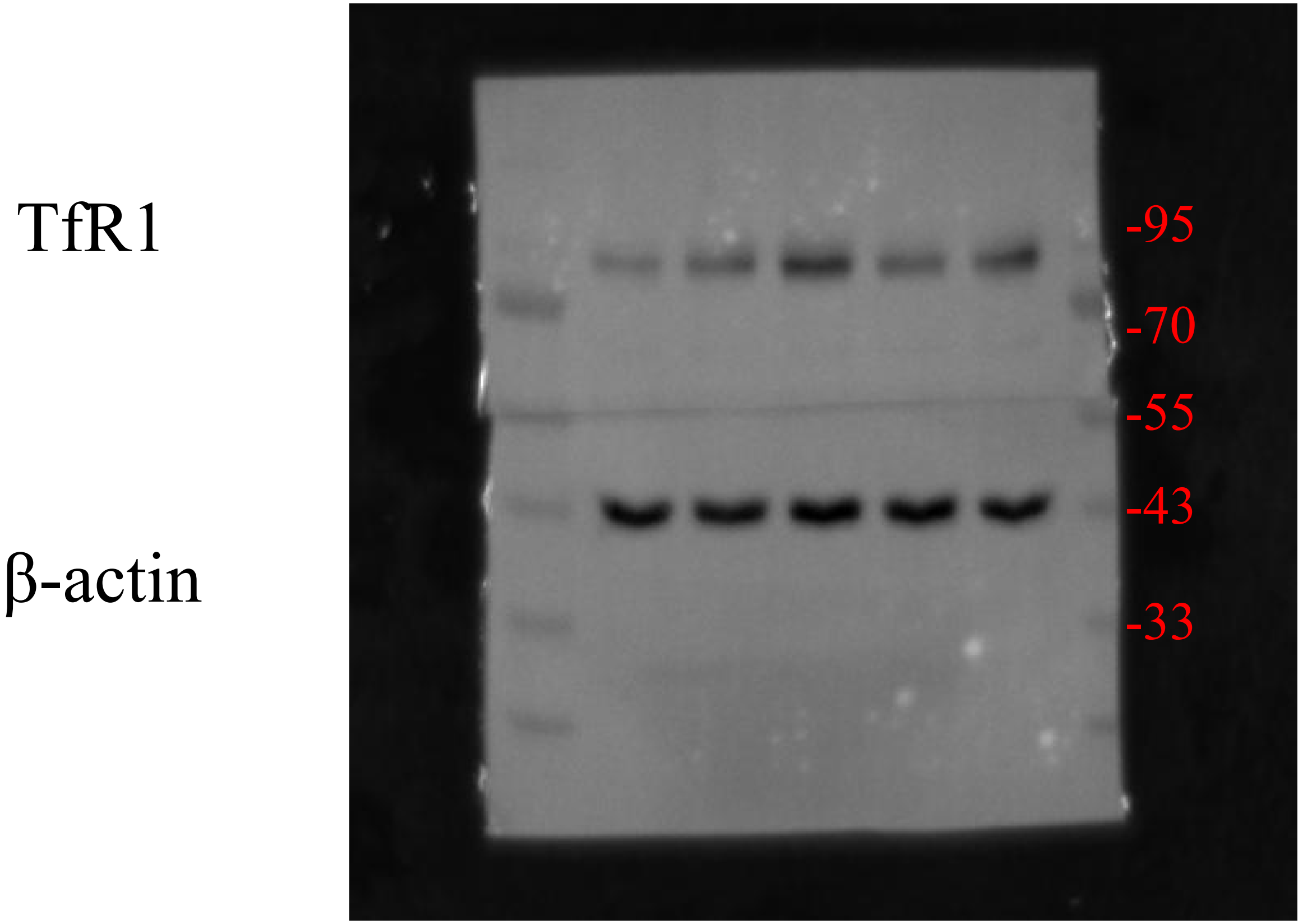

Additional file 2

TfR1

β-actin

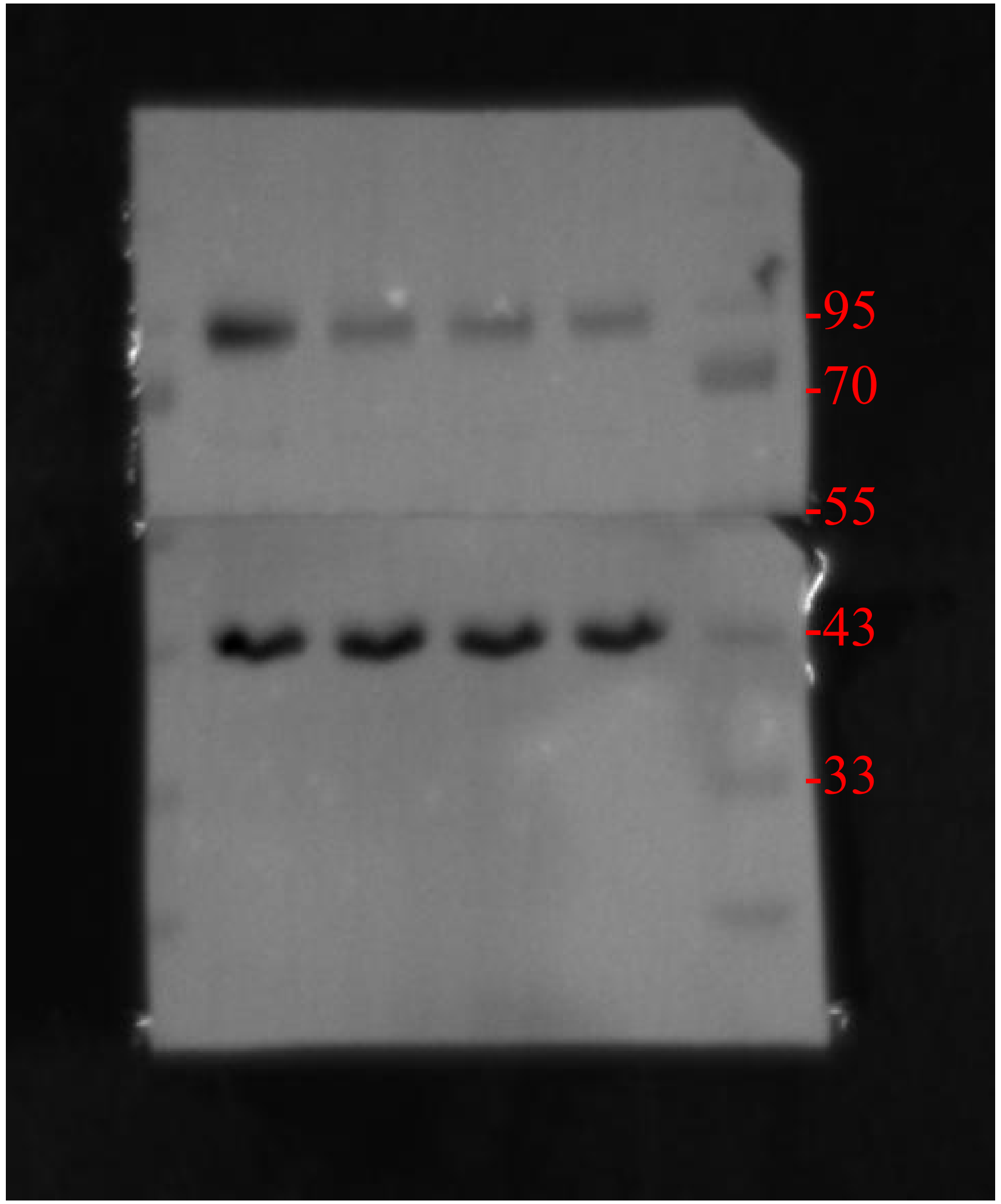

Supplement: Supplementary file 2 [file 12903_2026_9237_MOESM2_ESM.pdf]
