# Supplementary material for: 68Ga-citrate visualization study on ferroptosis through transferrin receptor 1 in periodontitis
Source: BMC Oral Health. 2026 Jul 13;26:1972. doi: 10.1186/s12903-026-09237-y (PMC13656373; doi:10.1186/s12903-026-09237-y)
Supplement: Supplementary file 3 [file 12903_2026_9237_MOESM3_ESM.pdf]

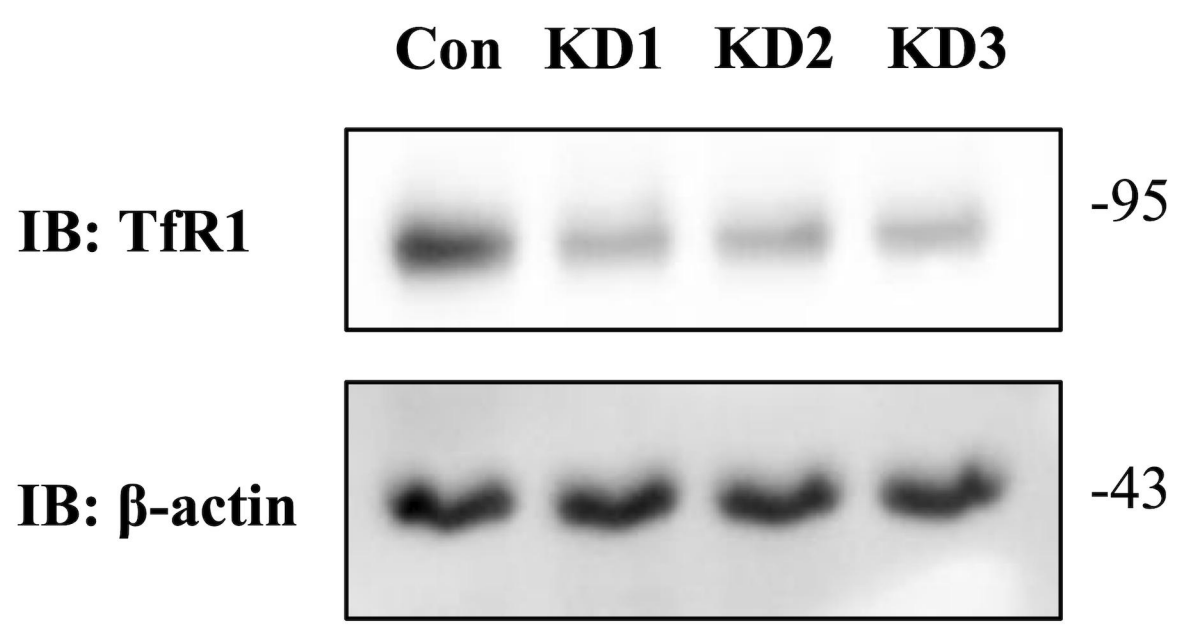

Additional file 2 TfR1 interference fragment. The TfR1 interference fragment was constructed and transfected MC3T3 cells. TfR1 protein expression by western blot was analyzed. KD, knock down; Con, Control group
